# Gene Polymorphisms Among *Plasmodium vivax* Geographical Isolates and the Potential as New Biomarkers for Gametocyte Detection

**DOI:** 10.3389/fcimb.2021.789417

**Published:** 2022-01-13

**Authors:** Anthony Ford, Daniel Kepple, Jonathan Williams, Gabrielle Kolesar, Colby T. Ford, Abnet Abebe, Lemu Golassa, Daniel A. Janies, Delenasaw Yewhalaw, Eugenia Lo

**Affiliations:** ^1^ Bioinformatics and Genomics, University of North Carolina, Charlotte, NC, United States; ^2^ Biological Sciences, University of North Carolina, Charlotte, NC, United States; ^3^ School of Data Science, University of North Carolina at Charlotte, Charlotte, NC, United States; ^4^ Department of Parasitology, Ethiopian Public Health Institute, Addis Ababa, Ethiopia; ^5^ Aklilu Lemma Institute of Pathobiology, Addis Ababa University, Addis Ababa, Ethiopia; ^6^ Tropical and Infectious Disease Research Center, Jimma University, Jimma, Ethiopia; ^7^ School of Medical Laboratory Sciences, Faculty of Health Sciences, Jimma University, Jimma, Ethiopia

**Keywords:** *Plasmodium vivax*, gametocyte detection, molecular biomarkers, genetic differentiation, malaria transmission

## Abstract

The unique biological features of *Plasmodium vivax* not only make it difficult to control but also to eliminate. For the transmission of the malaria parasite from infected human to the vector, gametocytes play a major role. The transmission potential of a malarial infection is inferred based on microscopic detection of gametocytes and molecular screening of genes in the female gametocytes. Microscopy-based detection methods could grossly underestimate the reservoirs of infection as gametocytes may occur as submicroscopic or as micro- or macro-gametocytes. The identification of genes that are highly expressed and polymorphic in male and female gametocytes is critical for monitoring changes not only in their relative proportions but also the composition of gametocyte clones contributing to transmission over time. Recent transcriptomic study revealed two distinct clusters of highly correlated genes expressed in the *P. vivax* gametocytes, indicating that the male and female terminal gametocytogeneses are independently regulated. However, the detective power of these genes is unclear. In this study, we compared genetic variations of 15 and 11 genes expressed, respectively, in the female and male gametocytes among *P. vivax* isolates from Southeast Asia, Africa, and South America. Further, we constructed phylogenetic trees to determine the resolution power and clustering patterns of gametocyte clones. As expected, *Pvs*25 (PVP01_0616100) and *Pvs*16 (PVP01_0305600) expressed in the female gametocytes were highly conserved in all geographical isolates. In contrast, genes including 6-cysteine protein *Pvs230* (PVP01_0415800) and upregulated in late gametocytes *ULG8* (PVP01_1452800) expressed in the female gametocytes, as well as two CPW-WPC family proteins (PVP01_1215900 and PVP01_1320100) expressed in the male gametocytes indicated considerably high nucleotide and haplotype diversity among isolates. Parasite samples expressed in male and female gametocyte genes were observed in separate phylogenetic clusters and likely represented distinct gametocyte clones. Compared to *Pvs*25, *Pvs230* (PVP01_0415800) and a CPW-WPC family protein (PVP01_0904300) showed higher expression in a subset of Ethiopian *P. vivax* samples. Thus, *Pvs230*, *ULG8*, and CPW-WPC family proteins including PVP01_0904300, PVP01_1215900, and PVP01_1320100 could potentially be used as novel biomarkers for detecting both sexes of *P. vivax* gametocytes in low-density infections and estimating transmission reservoirs.

## Introduction

*Plasmodium vivax* malaria is a neglected tropical disease, despite being more geographically widespread than other forms of malaria (Organization, 2018) and causes 132–391 million clinical infections each year worldwide (Price et al., 2007). *Plasmodium vivax* infections are most common in Southeast Asia and South America, with the Southeast Asian isolates being the most genetically diverse (Rougeron et al., 2020). Compared to *P. falciparum*, *P. vivax* has a broader temperature tolerance and an earlier onset of gametocyte development, enabling the parasites to spread through diverse climates (Elgoraish et al., 2019) and making them more difficult to control and eliminate (Lo et al., 2017). Although *P. vivax* infection is relatively benign, patients may experience similar clinical symptoms as *P. falciparum*, such as inflammatory responses, fever, and chills (Dayananda et al., 2018). The epidemiology of *P. vivax* malaria is further complicated by the parasite’s unique ability to form dormant-stage hypnozoites in the host liver cells, resulting in recurrent relapse infections from weeks or months to years later (White, 2011; Chu and White, 2016). Hypnozoites, which remain arrested in the liver for weeks to years, can be activated to cause new blood-stage infections and these features have substantially impacted progress in malaria control, especially in countries that are approaching elimination by providing parasite reservoirs for transmission at any time (Robinson et al., 2015; Lawpoolsri et al., 2019; Taylor et al., 2019).

A critical stage in *P. vivax* transmission is the development of gametocytes, known as gametocytogenesis, from trophozoites in the erythrocytic cycle. This allows *Plasmodium* species to be taken up by a mosquito vector, undergo sexual reproduction in the mosquito gut, and transmit to a new human host. Indeed, in the mosquito midgut, parasites can differentiate into their sexual forms, the female macrogametes and male microgametes. Gametocyte commitment is largely based on stress factors including high parasitemia, parasite strains, red blood cell density, anemia, drug treatments, and host immune responses (Smalley and Brown, 1981; Trager and Gill, 1992; Gautret et al., 1996; Talman et al., 2004; Drakeley C et al., 2006; Koepfli et al., 2015). Changes in temperature, pH, and host age can also stimulate gametogenesis in the human host (Bousema and Drakeley, 2011). When gametocytes are taken up during a mosquito’s blood meal, a number of factors including temperature, oxygen and carbon dioxide concentration, and pH can contribute to the maturation of gametocytes inside the mosquito midgut and salivary glands (Sinden, 1997). Other mosquito-derived factors such as xanthurenic acid can together activate transformation of gametocytes to male microgamete and female macrogamete within 5-10 minutes in the mosquito midgut. Although no clinical symptoms are experienced in the human hosts during gametocytogenesis, this developmental stage is critical for sexual reproduction in the mosquitoes and subsequent development of sporozoites that can infect other new human hosts. There is considerable variation in the development time of gametocytes amongst different human *Plasmodium* species, ranging from 7-10 days after the initial establishment of asexual parasites for *P. falciparum* (Gardiner and Trenholme, 2015) and 7-15 days for *P. vivax* (Bousema and Drakeley, 2011), indicating the need for reliable biomarkers for early-stage gametocyte detection.

A previous study has shown approximately 10% of *P. falciparum* and 60% of *P. vivax* infections have concurrent detectable low-density gametocytemia (Tadesse et al., 2017). Molecular tests for diminutive amounts of gametocytes rely on reverse-transcription polymerase chain reaction (RT-PCR) to amplify RNA transcripts of gametocyte-specifically expressed genes. Quantitative RT-PCR of targeted RNA transcripts reveals high sensitivity in detecting gametocytes of considerably low densities. For example, there are more than 106 copies of 18S rRNA transcripts per cell but only 5 copies of 18S rRNA gene per genome (Nishimoto et al., 2008). The production of high transcript copies in the parasite cells allow for greater detection limits. For *P. vivax*, *Pvs*25 and *Pvs*16, genes specific to the female gametocytes, are the two conventional gene markers for gametocyte detection (Wampfler et al., 2013). One gametocyte roughly corresponds to four *Pvs*25 transcripts per cell (Koepfli et al., 2015), and *Pvs*25 can detect from approximately 0.34 gametocytes per microliter of blood from *P. vivax* patients in Papua New Guinea (Wampfler et al., 2013) to 2 gametocytes per microliter of blood from *P. vivax* patients in Ethiopia (Tadesse et al., 2017). The number of *Pvs*25 gene transcript copies detected by qRT-PCR directly correlated with the number of mature gametocytes as well as the overall parasite densities (Bharti et al., 2006; Bousema and Drakeley, 2011) and showed a nearly normal distribution with a mean of 1.2×10^7^ copies/µL (ranging from 1.1 to 4.8×10^8^ copies/µL) blood among symptomatic *P. vivax* patients in northwestern Brazil (Lima et al., 2012). Such low gametocyte densities make them extremely difficult to be detected by microscopy and highlight the importance of sensitive molecular biomarkers in malaria-endemic regions.

Gametocytes are generally detected in ~20% of the infections among adults (Bousema and Drakeley, 2011), but at much higher proportions in children under the age of 12 (Nacher et al., 2004; Olliaro et al., 2016). Yet, gametocytemia in adults is up to 20-fold higher than in children (Dixon et al., 2008; Reece et al., 2009). In areas with low transmission, submicroscopic gametocytes could be hidden reservoirs for parasites with high proportions of infectious gametocytes (Hofmann et al., 2018). In Ethiopia, symptomatic *P. vivax* infections are nearly four times more infectious than asymptomatic ones (Tadesse et al., 2018). A recent study of 26 P*. vivax* samples from Cambodian patients indicated that the expression profile of 21 predicted gametocyte genes were clustered in two distinct groups (Kim et al., 2019). One group includes *Pvs*25, *ULG*8, gametocyte developmental protein 1, guanylate kinase, *HMGB*1, and five CPW-WPC proteins that associate with intracellular trafficking and histone remodeling in the female gametocytes. The other group includes *Pvs*47, *Pvs*48/45. *Hap*2, the gamete egress and sporozoite traversal protein, s16, and three CPW-WPC proteins that associate with microtubular development in the male gametocytes. It remains unclear if these male and female gametocyte genes show higher expression than the conventional marker *Pvs*25 and offer high detectability of total gametocyte densities (i.e., both male and female gametocytes). The detection of total gametocytes allows for robust gametocyte sex-ratio estimates in field studies given their stability under suboptimal conditions (Meerstein-Kessel et al., 2018). Gametocytes in low density infections can initiate transmission at any time, increasing the need to utilize reliable biomarkers for their detection and control. At the genomic level, polymorphisms in gametocyte-specific genes may provide information of parasite reservoirs that are transmitted from infected humans to mosquitoes, offering new insights into transmission bottlenecks in vectors. Genetic polymorphisms in gametocyte genes including *Pvs*25, *Pvs*28, *Pvs*48/45, and *Pvs*230 will also have important implications on their potential use and effectiveness as transmission blocking vaccine (TBV) candidates. Therefore, there are two key objectives in this study. First, genetic variations of 17 and 11 genes that represent female and male gametocytes, respectively, were compared among *P. vivax* isolates from Southeast Asia, East Africa, and South America based on whole genome sequence data. We constructed phylogenetic trees to determine clustering patterns with the goal of identifying novel DNA biomarkers with high differentiative power for gametocyte clones. Second, the expression levels of several female and male gametocytes were compared among a subset of Ethiopian *P. vivax* isolates based on transcriptomic data with the goal of identifying highly sensitive RNA biomarkers for both sexes of *P. vivax* gametocytes.

## Materials and Methods

### Data Collection

Whole blood samples were collected from 22 P*. vivax*-infected patients in Jimma, southwestern Ethiopia between September and November of 2016. We used Lymphoprep/Plasmodpur-based protocol to deplete the white blood cells and enrich the red blood cell pellets prior to DNA and RNA extractions. Genomic DNA was extracted from ~1 mL red blood cell pellets using the Quick-DNA Miniprep Kit (Zymo Research) following the manufacturer’s protocols. Only samples with monoclonal infections based on microsatellite genotyping were included for whole genome sequencing. These samples were collected from areas in Southern Ethiopia including Arbaminch, Badowacho, Halaba, and Hawassa (Auburn et al., 2019; Ford et al., 2020). An additional 39 samples of sequence data were obtained as FASTQ files from the European Nucleotide archive (ENA) that represent other East African countries including Uganda, Sudan, and Eritrea (Benavente et al., 2021). Sequence reads were mapped to the P01 reference genome (Auburn et al., 2016) available in Gene DB using BWA-MEMv2 (Li and Durbin, 2010; Langmead and Salzberg, 2012) with default settings. Only reads that were mapped to the reference were included and the quality of each of the aligned maps were assessed using FASTQC. The percentage coverage of the *P. vivax* reads was high for all samples (Ford et al., 2020). To provide a comparison of polymorphisms of our panel of gametocyte genes, we obtained an additional 72 P*. vivax* genomes including 50 genomes from southeast Asia (Cambodia and Thailand) and 22 from South America (Panama and Peru) (Pearson et al., 2016; Buyon et al., 2020). These genomes were obtained as FASTQ files from the ENA and Genbank. These genomes were also aligned to the P01 reference genome using BWA-MEMv2.

### SNP Discovery and Gene Diversity Analyses

Potential single nucleotide polymorphisms (SNPs) were identified using the genome analysis toolkit (GATK) version 4 (Van der Auwera and O’Connor, 2020) across all samples using the P01 reference genome. For the variant calling, we filtered the reads with the following scores: QD (quality by depth) less than 2.0, QUAL (read quality) less than 30, SOR (strand odds ratio) greater than 3.0, and MQ (map quality) score less than 40. From the high-quality SNPs, we obtained the consensus sequences of 28 gametocyte genes for further analyses. These 28 gametocyte genes were selected based on a previous study that showed the expression levels of several gametocyte markers were highly correlated with each other and clustered into two distinct groups (Kim et al., 2019). The microtubule-associated proteins including dynein, kinesin, and tubulin as well as few other male gametocyte genes were overrepresented in one cluster, while intracellular trafficking and histone remodeling genes were overrepresented in another. Therefore, in this study, a total of 17 female gametocyte genes including gametocytes ookinete surface protein *Pvs*25 (PVP01_0616100), gametocyte associated protein (PVP01_1403000), 6-cysteine protein *Pvs230* (PVP01_0415800), genes from the CPW-WPC family protein (PVP01_0820000, PVP01_0904300, PVP01_1003000, and PVP01_1223200), Guanylate kinase *PvGK* (PVP01_0727400), upregulated in late gametocytes *ULG8* (PVP01_1452800), gametocyte development protein 1 *PvGDV1* (PVP01_0734100), high mobility group protein *B1* (PVP01_1302200), and inner membrane complex protein 1j *ALV7* (PVP01_1128100), as well as 11 male gametocyte genes including 6-cysteine protein *P47 and P48/45* (PVP01_1208000, PVP01_1208100), gamete egress sporozoite traversal protein *GEST* (PVP01_1258000), sexual stage antigen s16 (PVP01_0305600), male gamete fusion factor HAP2 (PVP01_0814300), and genes from the CPW-WPC family protein (PVP01_1215900, PVP01_1119500, and PVP01_1320100) were analyzed. To compare genetic diversity of the 28 target gametocyte genes, we combined each of the consensus sequences into single fasta file and used MAFFT v.7 default settings for sequence alignment. Both the nucleotide diversity (pi) and haplotype diversity (Hd) of these genes across all 131 samples were calculated using DnaSP (Rozas et al., 2017). The Pairwise-Deletion method was used for calculation and gaps were excluded in each pairwise comparison.

### Phylogenetic Tree Reconstructions and Gene Network

To compare resolution power and genetic clustering pattern among the gametocyte genes, we constructed phylogenetic trees using Molecular Evolutionary Genetics Analysis (MEGA X) (Kumar et al., 2018). We selected the top five male and female gametocyte genes that revealed the highest haplotype and nucleotide diversity scores across all geographic locations. *Pvs*25 (PVP01_0616100) was used as a baseline for comparing phylogenetic resolution. For phylogenetic tree reconstructions, we first determined the best DNA substitution model for each of the genes. The maximum likelihood fits of 24 different nucleotide substitution models including the general time reversible model (TR), the Hasegawa-Kishino-Yano (HKY), the Tamura-Nei model (TN93), the Tamura 3-parameter model (T92), the Kimura 2-parameter model (K2), and the Jukes Cantor model (JC) was assessed by MEGA where the initial trees were automatically selected using the Neighbor-Join algorithms to a matrix of pairwise distances that were estimated using the Maximum Composite Likelihood approach. The best substitution model for each gene dataset was selected using a combination of the Bayesian Information Criterion (BIC), corrected Aikake Information Criterion (AICc), and log likelihood scores. Models with the lowest AICc were determined as the best substitution model for the gene. Using the optimal model, we constructed maximum likelihood phylogenetic trees in MEGA X. For the CPW-WPC family protein genes PVP01_1320100 and PVP01_1215900, the optimal model was the Kimura 2-Parameter model that is gamma distributed with invariant sites. For *PvULG8* (PVP01_1452800), we used the Hasegawa-Kishino-Yano substitution model that is gamma distributed with invariant sites. For *PvGDV1* (PVP01_0734100) and *Pvs230* (PVP01_0415800), we used the Tamura Nei substitution model that is gamma distribution with invariant sites. For each gene analysis, 100 bootstraps were performed to assess confidence of the genetic relationships among samples. We expanded on the resolving power in our phylogenetic trees by also constructing a gene transmission network using Strainhub (de Bernardi Schneider et al., 2020) for genes that showed clear genetic clustering among samples. Strainhub is a tool for creating gene flow networks using phylogenetic data and geographical metadata. The gene flow network was generated using the locations of our samples and calculating the source hub ratio (SHR) for each location. Numbers close to 1 represent places that are the sources of the transmissions, numbers close to 0.5 represent places that are the hubs through which transmissions pass and numbers that are close to 0 represent the sinks of the transmissions, places that are recipients.

### Genetic Distance and Selection Analyses

To validate the phylogenetic trees, we assessed the degree of genetic differentiation among geographical isolates for the targeted genes by calculating an Analysis of Molecular Variance model (AMOVA) and pairwise fixation indices (*F*_ST_ statistic) between samples using Arlequin ver 3.5.2.2 (Excoffier and Lischer, 2010). A global locus by locus AMOVA, with 1000 permutations, was constructed using the gene sequence data from all 131 samples and variations within and between countries were estimated. In addition, a matrix of *F*_ST_ values, using 100 permutations and a significance level of 0.05, was measured to indicate the level of population divergence, with values ranging from 0 (no evidence of population divergence) to 1 (completely isolated). *P*-values in both the AMOVA and pairwise *F*_ST_ matrix were calculated to assess the level of significance. For each country, we also tested for positive selection among the gametocyte genes by the codon-based Z-test implemented in MEGA X. The Nei-Gojobori method and a bootstrap procedure of 100 replicates were conducted. To validate the results of Z-test, we further estimated the Tajima’s D statistic using Tajima’s D test and D, D^*^, F, and F^*^ values using Fu and Li’s tests implemented in DnaSP (Rozas et al., 2017).

### Gametocyte Gene Expression Levels

To identify highly sensitive RNA biomarkers for male and female gametocytes, we examined the expression level of several male and female gametocyte genes and compared such with the standard *Pvs*25 in the a subset of 10 P*. vivax* samples from Ethiopia. A total of 10mL whole blood was preserved into sodium heparin from microscopy-confirmed *P. vivax* patients at hospitals in Jimma, Ethiopia, who had a minimum of 4,000 parasites/µL parasitemia and had not received prior antimalarial treatment. Scientific and ethical clearance was obtained from the institutional review boards of Jimma University, Ethiopia, and The University of North Carolina, Charlotte, USA. Written informed consent/assent for study participation was obtained from all consenting heads of households, parents/guardians (for minors under 18 years old), and from individuals who were willing to participate in the study. Upon collection, samples were cryo-preserved with 50% glycerolyte and stored in liquid nitrogen until *in vitro* culture. Prior to culture, samples were thawed by adding 0.2V of 12% NaCl solution drop-by-drop followed by a 5-minute room temperature incubation. Ten-times volume of the 1.6% NaCl solution was then added drop-by-drop to the mixture and the samples were centrifuged at 1000 rcf for 10 minutes to isolate the red pellet. This process was repeated with 10x volume of the 0.9% NaCl. Following centrifugation, the supernatant was removed *via* aspiration, and 18mL of sterile complete IMDM per 1mL cryo mixture was added to each sample for a final hematocrit of 2%. 10% Giemsa thick microscopy slides were made to determine majority stage (**Supplementary File 1**) and duration of incubation, averaging 20-22 hours for majority trophozoites and 40-44 hours for majority ring. Samples were then incubated at 37°C in a 5% O₂, 5% CO₂ atmosphere to allow growth to the schizont stage.

*In vitro* maturation was validated through microscopic smears 17 hours after the initial starting time and subsequently checked every one to two hours. Cultured pellets were isolated *via* centrifugation and placed in 10x volume trizol for RNA extraction. RNA extraction was performed using Direct-zol RNA prep kit (Zymo Research) according to the manufacturer protocol, with two rounds of DNA digestion using the DNA-free kit (Zymo Research). Samples were analyzed with a nanodrop 2000 and RNA Qubit to ensure sample concentrations were above 150 ng total for library construction. For samples with no significant amount of DNA, RNA libraries were constructed using Illumina rRNA depletion library kits according to the manufacturer protocol. Sample reads were obtained using Illumina HiSeq 2x150bp configuration to obtain at least 35 million reads per sample. Sequence reads were aligned in HISAT2 to the P01 *P. vivax* reference genome and all human reads were filtered out using SAMtools. The alignment was mapped to the P01 reference annotation using the R package subread. Samples were then deconvoluted in CIBERSORTx based on *P. falciparum* homologs to obtain a transcription profile. Analyses of 25 targeted gametocyte genes including10 male and 15 female gametocyte genes were performed using DESeq2 to indicate expression levels among samples (see **Supplementary File 2** for accession numbers).

## Results

### Comparison of Nucleotide and Haplotype Diversity

**Figure 1:**
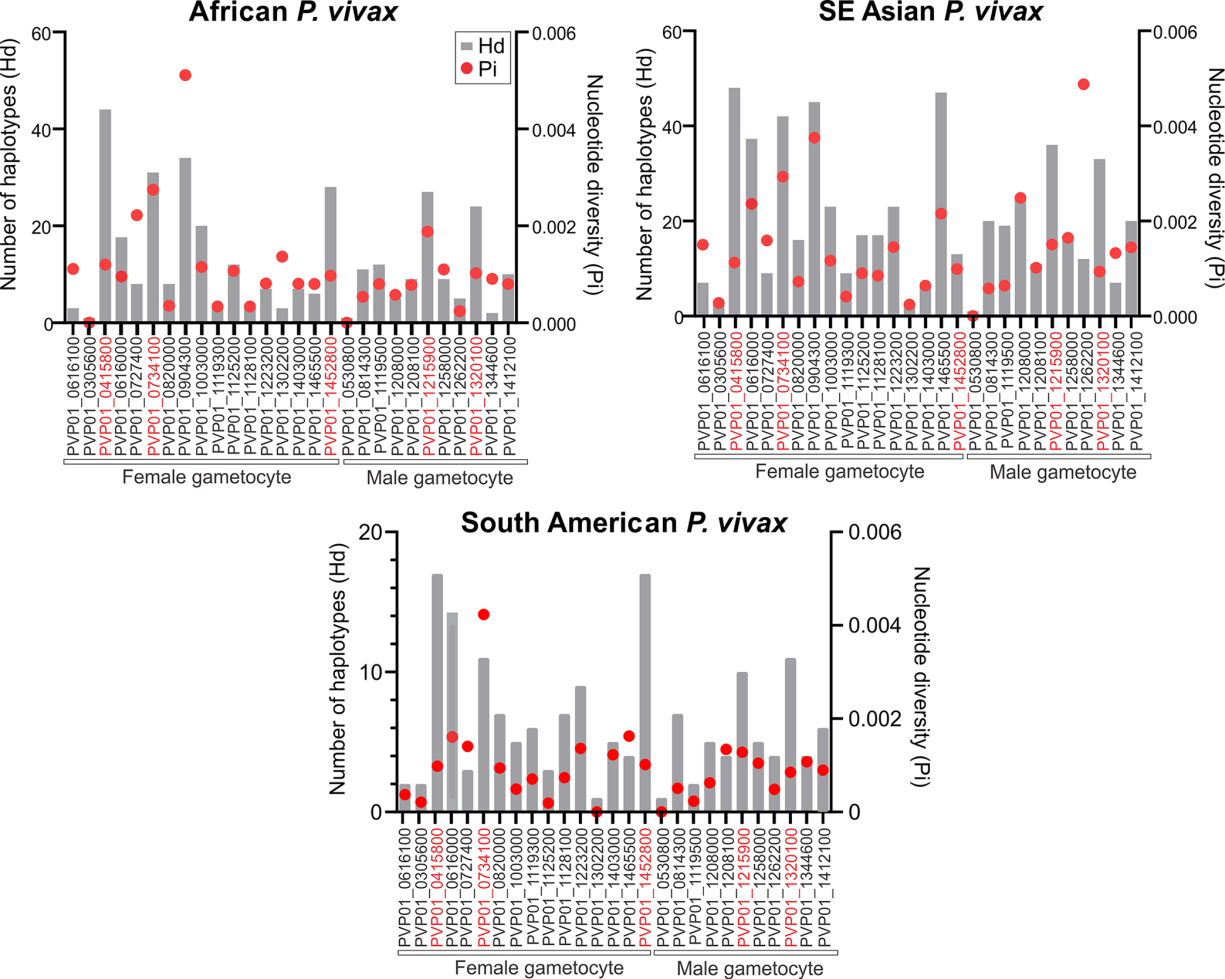
Plots comparing the haplotype diversity and nucleotide diversity scores of all 17 female and 11 male gametocyte genes including *Pvs*25 (PVP01_0616100) among isolates obtained from East Africa, Asia, and South America. The gray bar represents haplotype diversity and the red dots represent the corresponding nucleotide diversity scores. Across all geographical isolates, *Pvs230* (PVP01_0415800), *GDV1* (PVP01_0734100), CPW-WPC family protein genes PVP01_1215900 and PVP01_1320100, *ULG8* (PVP01_1452800), and *Pvs*28 (PVP01_0616000) were highly polymorphic compared to the other gametocyte genes.

For the East African (Ethiopia, Sudan, Eritrea, and Uganda) *P. vivax* isolates, *Pvs*25 (PVP01_0616100) had a haplotype diversity (Hd) of 0.58 and nucleotide diversity (Pi) of 1.39×10^-3^ (**Figure 1**; **Supplementary File 3**). Of the 17 female gametocyte genes, *Pvs230* (PVP01_0415800; Hd: 0.999 and Pi: 1.24×10^-3^), *GDV1* (PVP01_0734100; Hd: 0.965 and Pi: 2.86×10^-3^), and *ULG8* (PVP01_1452800; Hd: 0.939 and Pi: 1.01×10^-3^) were the most polymorphic compared to *Pvs*25 (**Figure 1**). Of the 11 male gametocyte genes, two CPW-WPC gamily protein genes PVP01_1215900 (Hd: 0.946 and Pi: 1.80×10^-3^) and PVP01_1320100 (Hd: 0.940 and 9.58×10^-4^) were most polymorphic (**Figure 1**). For the Southeast Asian *P. vivax*, *Pvs230* (PVP01_0415800; Hd: 0.998 and Pi:1.13×10^-3^), CPW-WPC gamily protein gene PVP01_0904300 (Hd: 0.995 and Pi: 3.75×10^-3^), and *ULG8* (PVP01_1452800; Hd: 0.998 and Pi: 2.16×10^-3^) had the highest levels of polymorphism among the 17 female gametocyte genes. Of the 11 male gametocyte genes, PVP01_1215900 (Hd: 0.985 and Pi: 1.51×10^-3^) and PVP01_1320100 (Hd: 0.963 and Pi: 9.36×10^-4^) were most polymorphic. *Pvs*25 in the Cambodian and Thailand *P. vivax* was more polymorphic than the Ethiopian (Hd: 0.67 and Pi: 1.5×10^-3^; **Figure 1**). and South American isolates (Hd: 0.247 and Pi: 3.74×10^-4^; **Figure 1**). Similarly, for the South American (Peru and Panama) *P. vivax*, *Pvs230* (Hd: 0.96 and Pi: 9.83×10^-4^) and *ULG8* PVP01_1452800 (Hd: 0.96 and Pi: 1.02×10^-3^) were the most polymorphic among the 17 female gametocyte genes, consistent to the pattern observed in other geographic regions (**Figure 1**). Of the 11 male gametocyte genes, two CPW-WPC gamily protein genes PVP01_1215900 (Hd: 0.896 and Pi: 1.28×10^-3^) and PVP01_1320100 (Hd: 0.896 and 8.54×10^-4^) were the most polymorphic (**Figure 1**). Compared to *Pvs*25 and *Pvs*230, the other TBV gene candidate *Pvs*28 (PVP01_0616000) showed high polymorphisms among all geographic isolates, whereas *Pvs*48/45 (PVP01_1208100) was relatively conserved (**Figure 1**). Therefore, these six genes including *Pvs230* (PVP01_0415800), *GDV1* (PVP01_0734100), CPW-WPC family protein genes PVP01_1215900 and PVP01_1320100, *ULG8* (PVP01_1452800), and *Pvs*28 (PVP01_0616000) were selected for further analyses using *Pvs*25 (PVP01_0616100) as a standard.

### Genetic Clustering Patterns and Resolving Power

**Figure 2:**
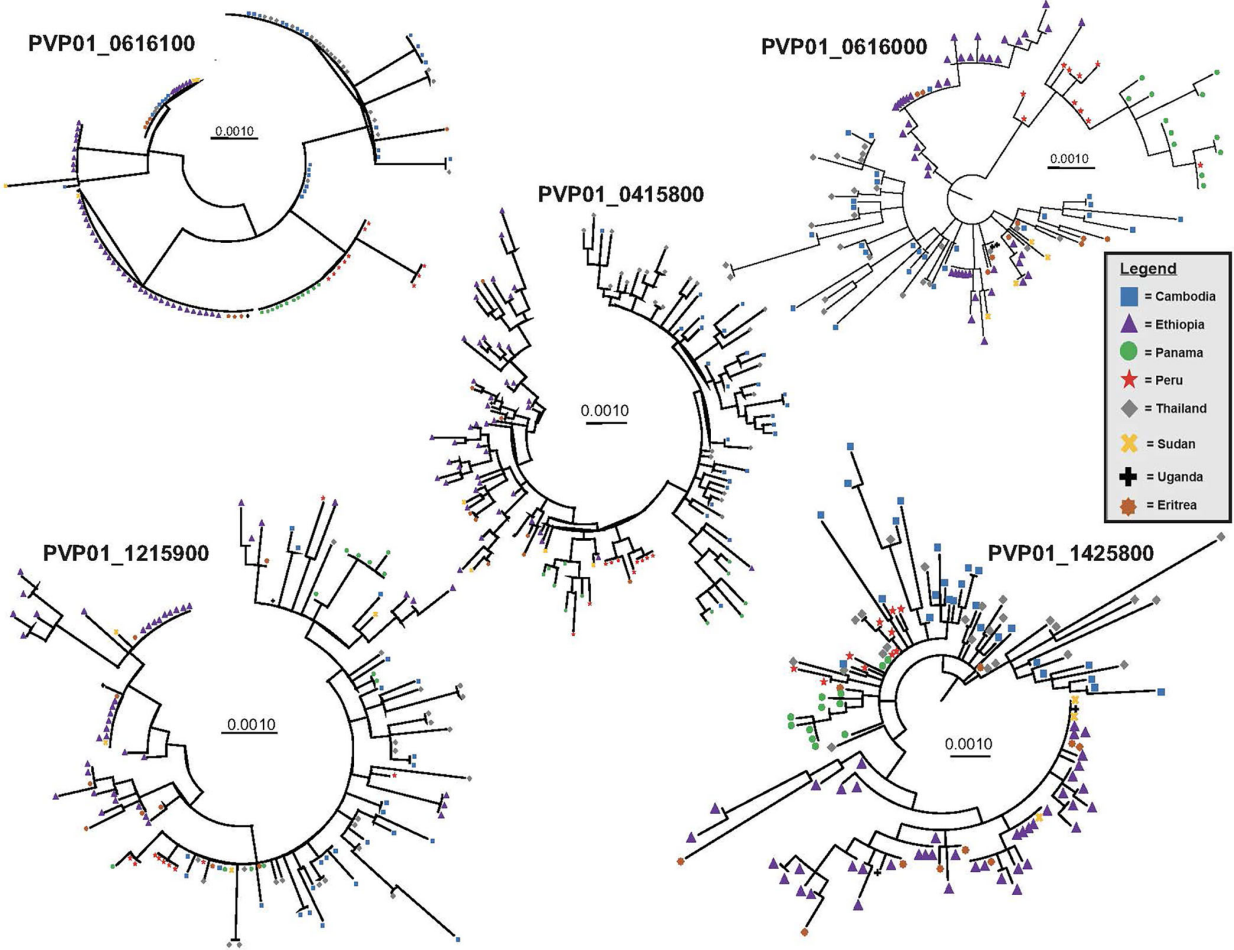
Phylogenetic trees for four highly polymorphic gametocyte genes *Pvs230* (PVP01_0415800), *Pvs*28 (PVP01_0616000), *ULG8* (PVP01_1452800), and CPW-WPC family protein gene PVP01_1215900 illustrating the resolving power of gametocyte clones as compared to the standard *Pvs*25 (PVP01_0616100). Countries (Cambodia, Ethiopia, Eritrea, Panama, Peru, Thailand, Sudan, and Uganda) of the same continent were clustered together without clear differentiation. Among all, *Pvs*230 (PVP01_0415800) and *PvULG*8 (PVP01_1452800) showed most clear genetic differentiation between continents.

For the three female (*Pvs230*, *GDV1*, and *ULG8*) and two male (PVP01_1215900 and PVP01_1320100 of the CPW-WPC gene family) gametocyte genes that showed high polymorphisms across the East African, Southeast Asian, and South American *P. vivax* isolates, the substitution model was first determined for each gene set prior to phylogenetic reconstructions. For genes PVP01_1215900 and PVP01_1320100, the Kimura two-parameter model with gamma distribution and invariant sites was selected as the best model based on the AICc and log likelihood scores (**Table 1**). For *Pvs25*, *GDV1*, and *Pvs*28, the Tamura 3 Parameter model was selected. For *ULG8*, the Hasegano-Kishino-Yano substitution model with gamma distribution and invariant sites was selected (**Table 1**). Most of the phylogenetic trees showed clear geographic differentiation between the Ethiopian and other geographical isolates (**Figure 2**), except for *GDV1* (PVP01_0734100) and CPW-WPC gene PVP01_1320100 where the Ethiopian *P. vivax* were mixed with the Southeast Asian and South American isolates (**Supplementary File 4**). In the *Pvs*25 (PVP01_0616100) phylogeny (**Figure 2**), four major clades were detected. The Ethiopian isolates were found in two of the clades, one of which contained also the Southeast Asian *P. vivax*. The South American samples were found exclusively in a separate clade. Within each clade, no resolution was detected among samples. In the *Pvs230* (PVP01_0415800) phylogeny (**Figure 2**), samples from Peru and Panama (South America) are clustered together sister to the Cambodian and Thailand (Southeast Asia) isolates. The Ethiopian *P. vivax* were genetically distant from these geographical isolates. Within each major clade, samples were well differentiated from one another. Likewise, in the *ULG8* (PVP01_1452800), *Pvs*28 (PVP01_0616000), and CPW-WPC gene PVP01_1215900 phylogenies (**Figure 2**), the Ethiopian *P. vivax* were clearly differentiated from the South American and Southeast Asian isolates. The South American and Southeast Asian isolates were well mixed with one another, though a small subclade containing only the Cambodian and Thailand samples was observed. For most genes, countries of the same continent were clustered together without clear differences. The resolving power of *Pvs230* (PVP01_0415800) and *ULG8* (PVP01_1452800) was the highest among all gametocyte genes (see **Supplementary File 5** for primer information of these two genes).

**Table 1:** Summary of the best DNA substitution model selected for each of the gametocyte genes and the metrics corrected AIC and log likelihood scores used in phylogenetic reconstructions.

Gene	Gene Description	Model Selection		
		Model	AICc	Log Likelihood
**PVP01_0415800**	6-cysteine protein P230, putative	Tamura Nei	34970.180	-17219.032
**PVP01_0734100**	Gametocyte development protein 1, putative GDV1	Tamura Nei	12213.975	-5840.786
**PVP01_1452800**	Upregulated in late gametocytes, putative ULG8	Hasegawa- Kishino-Yano	13204.928	-6337.308
**PVP01_1215900**	CPW-WPC family protein	Kimura 2 parameter	15092.227	-7283.982
**PVP01_1320100**	CPW-WPC family protein	Kimura 2 parameter	19448.261	-9462.261
**PVP01_0616000**	Ookinete surface protein P28, putative	Tamura 3 parameter	8054.830	-3764.160
**PVP01_0616100**	Ookinete surface protein P25	Tamura 3 parameter	5619.745	-2547.554

**Figure 3:**
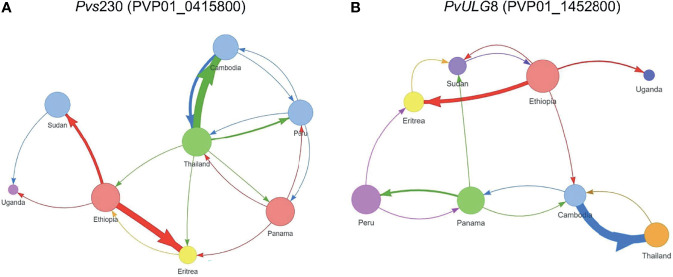
Source Hub Ratio (SHR) transmission networks that further expand the resolving power for **(A)** *Pvs*230 (PVP01_0415800) and **(B)** *PvULG*8 (PVP01_1452800). The size of each node is proportional to the node’s SHR value. Node colors were randomly assigned to each unique SHR value present in the plot to improve readability. Similarly, arrow colors are paired with the color of the node from which they begin to aid with interpretation. The weight of each arrow is proportional to the number of transitions between those two nodes. The position of nodes is arbitrary and is not equivalent to the position of sample sites in geographic space. More transitions were detected between African as well as between Asian countries, with few instances of gene flow from Asia to Africa. Ethiopia and Thailand had a Source Hub Ratio (SHR) of 0.6 and 0.625, respectively, meaning that these two countries are likely the source of infections. Parasite gene flow was detected the highest among the East African countries, e.g., from Ethiopia to Sudan and Eritrea, as well as from Thailand to Cambodia in Southeast Asia **(A)**. Similar pattern was observed in the *PvULG*8 (PVP01_1452800) based network **(B)**.

The AMOVA results showed that the variation observed between samples within each country was generally higher than that between countries (**Supplementary File 5**). Between countries, *Pvs25* (PVP01_0616100) and *Pvs28* (PVP01_0616000) have the greatest level of genetic differentiation (percent variation of 42% and 44%; *P*<0.05; **Supplementary File 5**), followed by *Pvs230* (PVP01_0415800) and *ULG8* (PVP01_1452800) with percent variations of about 32%. The pairwise *F*_ST_ values based on *Pvs230* (PVP01_0415800) and *ULG8* (PVP01_1452800) were on average 30%, consistent with their phylogenetic tree showing a much clearer distinction between countries than the other genes (**Figure 2**). Comparatively, *GDV1* (PVP01_1452800) and the CPW-WPC gene PVP01_1320100 had very low percentage variation between countries (percent variations of 15% and 18%, respectively) but much higher variations within each country (**Supplementary File 5**). Such low levels of genetic differentiation were consistent with their phylogenetic trees showing *P. vivax* from different countries in several clades without clear geographical distinction (**Supplementary File 3**). The gene transmission networks echoed the phylogenetic relationships. For instance, the network based on *Pvs230* (PVP01_0415800; **Figure 3A**) showed that Ethiopia and Thailand had a Source Hub Ratio (SHR) of 0.6 and 0.625, respectively, meaning that these two countries are likely the source of infections. Parasite gene flow was detected the highest among the East African countries, e.g., from Ethiopia to Sudan and Eritrea, as well as from Thailand to Cambodia in Southeast Asia (**Figure 3A**). Similar pattern was observed in the *PvULG*8 (PVP01_1452800) based network (**Figure 3B**).

### Signature of Positive Selection

Of the seven male and female gametocyte genes chosen, *ULG*8 (PVP01_1452800) was detected with significant positive selection by the codon-based Z test among *P. vivax* isolates in Cambodia, Thailand, Ethiopia, and Eritrea (*P*<0.01; **Table 2**). Other genes including *GDV1* (PVP01_1452800), *Pvs230* (PVP01_0415800), and the two CPW-WPC genes PVP01_1215900 and PVP01_1320100 were not detected with positive selection (**Table 2**). The negative Tajima’s D values detected in all the seven gametocyte genes indicated that these genes are not neutral.

**Table 2:** Positive selection test results based on codon-based Z test in MEGA X at 95% significance level.

Country	Positive selection *P*-value						
	Pvs230 PVP01_0415800	PvGDV1 PVP01_0734100	CPW-WPC protein gene PVP01_1215900	CPW-WPC protein gene PVP01_1320100	PvULG8 PVP01_1452800	Pvs25 PVP01_0616100	Pvs28 PVP01_0616000
**Cambodia**	0.482	1.000	1.000	1.000	0.001	1.000	0.128
**Thailand**	1.000	1.000	1.000	0.360	0.001	0.114	0.273
**Peru**	0.289	1.000	0.262	1.000	0.046	1.000	0.149
**Ethiopia**	0.440	1.000	0.363	0.239	0.002	0.051	0.314
**Panama**	0.027	1.000	1.000	1.000	0.227	1.000	0.179
**Eritrea**	0.369	1.000	1.000	1.000	0.001	0.042	1.000
**Sudan**	0.169	1.000	1.000	0.419	0.069	0.085	0.266
**Uganda**	0.356	1.000	0.229	0.038	0.042	1.000	1.000
**Tajima’s statistic**	-1.78	-1.658	-1.687	-2.117	-1.740	-1.045	-1.319

Evidence for potential positive selection was observed in PvULG8 (PVP01_1452800) among the Cambodian, Thailand, and Ethiopian P. vivax isolates.

### Expression of Female and Male Gametocyte Genes

**Figure 4:**
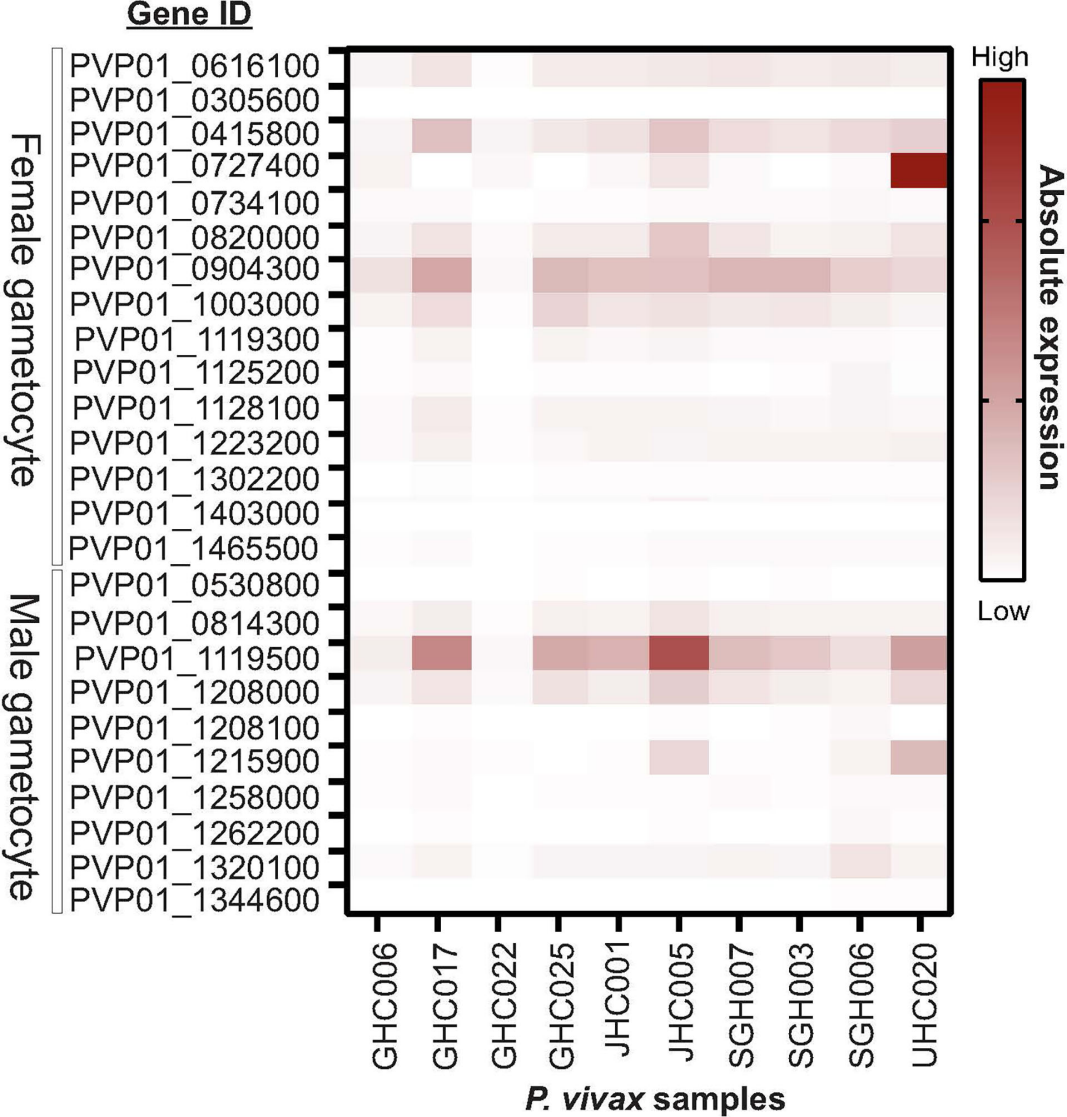
Heatmap plot illustrating the expression level of 15 male and 10 female gametocyte genes for 10 Ethiopian *P. vivax in vitro* samples. Out of the 10 samples, eight contained gametocytes based on *Pvs25* screening whereas the other two samples (GHC006 and GHC022) did not express *Pvs25*. Amongst the 25 gametocyte genes, two CPW-WPC genes PVP01_0904300 and PVP01_1119500 that represent male and female gametocytes, respectively, were most highly expressed compared to the standard *Pvs25* across all eight gametocyte-positive samples.

**Figure 5:**
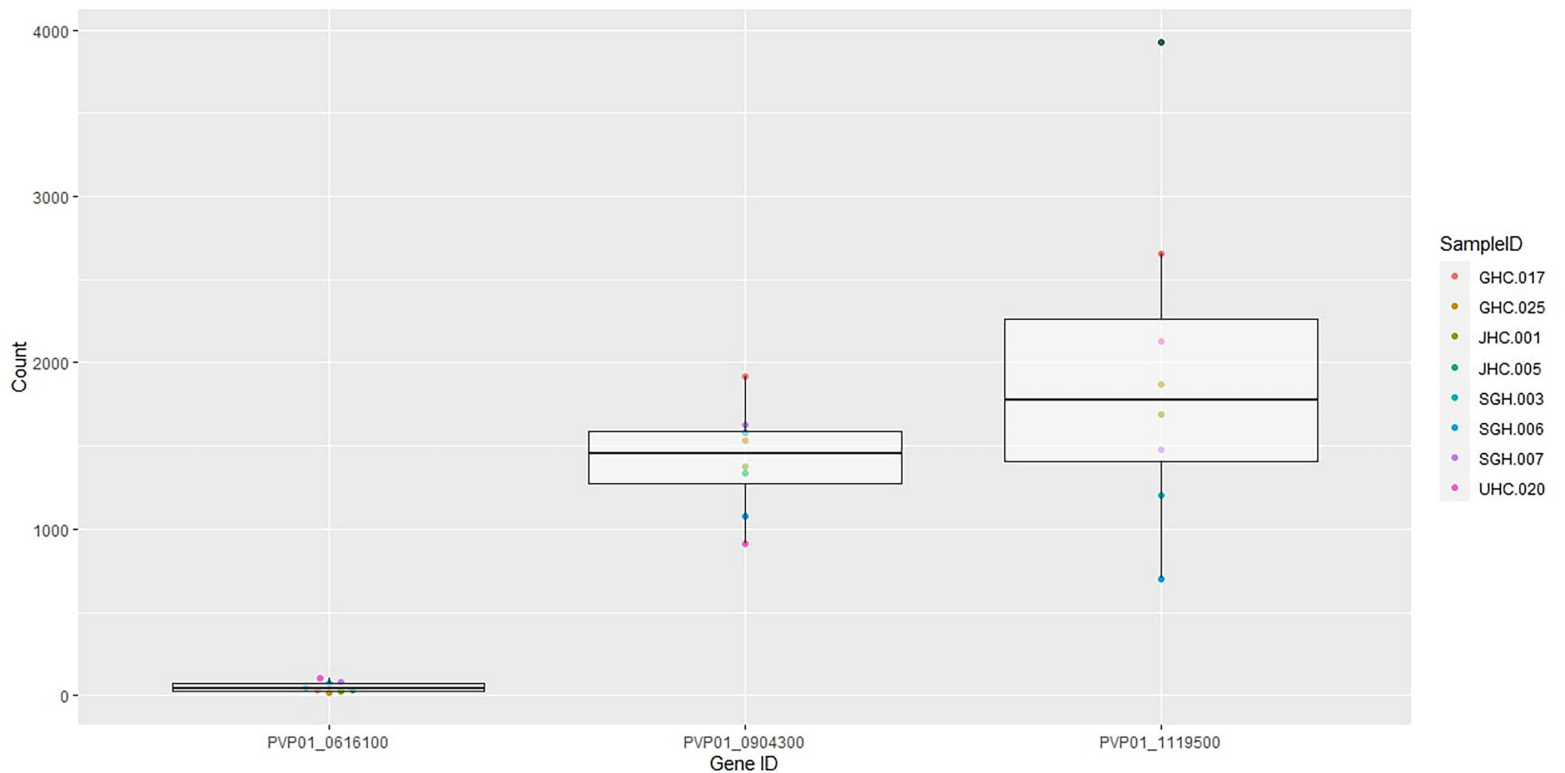
Boxplots of *Pvs*25 (0616100) and the two CPW-WPC genes PVP01_0904300 and PVP01_1119500 showing gene expression level differences in the eight gametocyte-positive samples.

Eight out of ten *in vitro* samples from Ethiopia contained submicroscopic gametocytes based on *Pvs*25 screening. Of these, two CPW-WPC genes PVP01_0904300 and PVP01_1119500 that represent the female and male gametocytes, respectively, were highly expressed (**Figure 4**) and consistently higher than *Pvs*25 across all samples (**Figure 5**). Apart from PVP01_0904300, another female gametocyte gene *Pvs230* (PVP01_0415800) also showed a relatively high level of expression. *Pvs230* (PVP01_0415800) and PVP01_0904300 also revealed high levels of haplotype and nucleotide diversity (**Figure 1**) and high resolving power to *P. vivax* geographical samples (**Figure 2**).

## Discussion

The transmission of *P. vivax* relies on the development of gametocytes from committed schizonts in the human hosts and uptake by the *Anopheles* mosquitoes. Gametocytes especially in low-density and/or submicroscopic infections are hidden parasite reservoirs that can initiate transmission at any time. Thus, it is critical to utilize reliable biomarkers for their detection and effective malaria control intervention. *Pvs*25 and *Pvs*16 are two commonly used biomarkers for *P. vivax* gametocyte detection (Wampfler et al., 2013). However, they represent only female gametocytes and could underestimate the total gametocytes present in an infection. The proportions of infectious gametocytes as well as the ratio of male to female gametocytes can vary across infections, and in turn, determine parasitemia in the mosquito and the transmission potential (Tadesse et al., 2019). Gametocyte sex ratio positively correlates with gametocyte density with generally higher proportions of microgametocytes especially in low-density infections, which could result in optimal fertilization and higher gametocyte densities (Schall, 2000; Talman et al., 2004). Additionally, the ability to accurately detect male and female gametocytes will enable us to understand how anti-malarial drugs affect gametocyte production and transmission in natural infections (Henry et al., 2019) and develop transmission blocking therapies. In this study, we identify one male gametocyte CPW-WPC protein gene PVP01_1119500 and two female gametocyte genes *Pvs230* (PVP01_0415800) and CPW-WPC gene PVP01_0904300 that showed high expression relative to *Pvs*25. Both PVP01_0904300 and PVP01_1119500 belong to the CPW-WPC gene family, and *Pvs230* (PVP01_0415800) is a surface protein of *P. vivax* female gametocytes.

Better understanding of genetic polymorphisms in gametocyte genes have important implications on their potential in the development of transmission blocking vaccine candidates. *Pvs*25 is a highly expressed but conserved female gametocyte gene. It is one of the leading candidates for malaria TBV based on its high immunogenicity observed in animal model studies and high conservation among *P. vivax* isolates from endemic regions (Feng et al., 2011; Chaurio et al., 2016). In endemic regions of the Amazon in South America, *Pvs*25 was shown to be conserved among *P. vivax* isolates (Chaves et al., 2019). *Pvs*25 has been reported with positive selection in a previous study (Chaurio et al., 2016). The present study indicated that *Pvs*25 is less polymorphic than other female and male gametocyte genes and is not neutral. *P. vivax* isolates from the same country were more closely related or genetically similar than from different countries. These findings suggested that selection may have contributed to the conserved nature of this gene.

*Pvs*48/45, a male gamete fertilization factor, is another main transmission blocking vaccine candidate (Feng et al., 2015). Our analyses indicated a relatively conserved nature of this gene among global isolates, agrees with a previous study that indicated low levels of genetic diversity in *Pvs48/45* among 200 P*. vivax* isolates from temperate and subtropical populations in China (Feng et al., 2015). Of the 14 SNPs, 11 were nonsynonymous with an overall nucleotide diversity of 0.0012 across the isolates. At the continental level, *F*_ST_ values based on *Pvs*48/45 widely ranged from 0.34 to 0.90 among samples from several countries in Asia, America, and Oceania, with an overall *F*_ST_ of 0.665 (Feng et al., 2015). Though earlier studies of isolates from China showed *Pvs*48/45 undergoing potential positive selection with the distribution of polymorphic sites concentrated in domain II of the gene (Feng et al., 2015), the present study did not detect significant positive or non-neutral selection in this gene. High levels of antibodies against Pfs48/45 and Pfs47 were previously reported in *P. vivax* endemic regions (Anthony et al., 2007). Immuno-epidemiological studies of the parasite sexual stage antigens including Pvs48/45 and Pvs230 showed that antibodies against these antigens are present in people living in *P. vivax* endemic areas and associated with transmission blocking activities (Bousema et al., 2006; Drakeley CJ et al., 2006; Bousema et al., 2010; Feng et al., 2015; Jones et al., 2015). It is possible that these gene antigens illicit host antibody responses and impose selection, leading to scarce genetic polymorphisms in the parasite populations.

Gametocyte production is critical for sexual reproduction of the parasite and subsequent transmission to a new host. It is essential to identify gametocyte clones and the origin of transmission to effectively identify transmission reservoirs in both symptomatic and asymptomatic infections. Prior studies have demonstrated that low density *P. vivax* gametocytes in asymptomatic carriers significantly contribute to transmission and genetic diversity (Bousema et al., 2014; Abdelraheem et al., 2018). Lower genetic diversity in *P. vivax* populations is correlated with lower transmission frequencies and low amounts of imported parasites, resulting in fewer clones (Nguitragool et al., 2017; Kepple et al., 2021). Our findings showed that two female gametocyte genes *Pvs*230 (PVP01_0415800) and *ULG*8 (PVP01_1425800) were highly polymorphic compared to *Pvs25*, which are potential DNA markers for determining the source of *P. vivax* gametocytes and differentiating gametocyte clones within and between hosts. Previous studies indicated limited polymorphism in *Pvs*230, making this gene a potential candidate of malaria transmission blocking vaccine (Doi et al., 2011). In a study involving 112 full length *Pvs*230 sequences of *P. vivax* isolates from regions in South America, Southeast Asia, Indo-West Pacific, and Madagascar, polymorphism of this gene was much lower than the bloodstage antigen genes (Doi et al., 2011), likely due to the fact that bloodstage antigens are targets of host antibody responses and high genetic diversity in the antigen genes would facilitate host immune response evasion (Doi et al., 2011). Our findings did not support the conservative nature of *Pvs*230 among different geographical isolates. Instead, we detected high genetic differentiation of *Pvs230* among *P. vivax* from the same geographic region. Our transmission network based on *Pvs230* further showed as high as 13 transitions between Cambodia and Thailand, a high level of gene flow within Southeast Asia. On the other hand, the limited gene flow among the continental isolates may explain the differentiation of *Pvs230*. No significant positive selection was detected in *Pvs230* in this study, likely because this protein located on the surface of gametocytes is involved in gamete recognition and fertilization and could be highly divergent among parasite populations (Tsaur et al., 2001; Palumbi, 2009; Doi et al., 2011). Nevertheless, the high polymorphisms observed in *Pvs230* makes this gene a less viable candidate for TBV.

The CPW-WPC proteins were previously shown to be expressed on the surface of *P. falciparum* and *P. berghei* ookinetes and may play a role in transmission by the mosquito (Kangwanrangsan et al., 2013; Rao et al., 2016). In Thailand, CPW-WPC genes were detected with significant positive selection likely due to their role in mosquito transmission (Diez Benavente et al., 2017), which may raise concerns on the reliability of using CPW-WPC protein genes as biomarkers. *PvULG*8 (PVP01_1452800) was detected with positive selection in the Cambodian, Thailand, and Ethiopian *P. vivax*. The ortholog *PfULG*8 is a member of the *P. falciparum* gene family encoding the CPW-WPC proteins. An integrated transcriptomic and proteomic analysis of *P. falciparum* gene expression showed that transcripts from nine CPW-WPC genes predominantly accumulate in female gametocytes and are subjected to translational repression (Lasonder et al., 2016; Siciliano et al., 2017). Though these transcripts are produced in gametocytes, they are translated only in the mosquito parasite stages (Siciliano et al., 2017). Further studies examining diversity of CPW-WPC genes, especially PVP01_0904300 and PVP01_1119500, are needed to understand the full potential of these two genes for gametocyte detection. In this study, the transcriptomic data were based on a small number of Ethiopian isolates. In the future, it is important to correspond SNPs obtained from gDNA with SNPs on expressed transcripts in gametocytes and other epidemiological features such as the infectiousness and host immune responses of broader samples in order to understand better the functional significance of polymorphisms detected at the DNA level.

## Conclusion

Conventional methods of detecting *P. vivax* gametocytes may underestimate the level of transmission reservoirs. A new approach is needed to better detect and monitor the transmission potential especially in countries approaching malaria elimination. The present study examined polymorphisms and resolving power of 28 male and female gametocyte genes through phylogenetic and selection testing. Given high genetic differentiation and clear clustering of gametocyte clones by *Pvs230* (PVP01_0415800) and *PvULG8* (PVP01_1452800), these two genes could provide a better detection method of identifying transmission reservoirs and source of transmission for symptomatic and asymptomatic malarial infections. Transcriptomic data of a few Ethiopian *P. vivax* samples showed a high expression of *Pvs230* (PVP01_0415800) as compared to *Pvs*25, suggesting its potential as a sensitive RNA marker for detecting low-gametocytemia infections. It is noted that *PvULG8* was detected with positive selection in the Southeast Asian and African *P. vivax*, and the underlying reason warrants further investigation. Future studies on gametocyte reservoirs especially in asymptomatic and/or low-density infections using *Pvs230* and *PvULG8* may shed light on the importance of these genes as biomarkers in the detection of gametocytes.

## Data Availability Statement

The datasets presented in this study can be found in online repositories. The names of the repository/repositories and accession number(s) can be found in the article/**Supplementary Material**.

## Ethics Statement

The studies involving human participants were reviewed and approved by the Scientific and ethical clearance was obtained from the institutional review boards of Jimma University, Ethiopia, and The University of North Carolina, Charlotte, USA. Written informed consent/assent for study participation was obtained from all consenting heads of households, parents/guardians (for minors under 18 years old), and from individuals who were willing to participate in the study. The patients/participants provided their written informed consent to participate in this study.

## Author Contributions

Conceptualization, LG, DY, and EL. Resources, DJ, DY, and EL. Conduct experiment and data analyses, AF, DK, JW, GK, CF, and AA. Writing—original draft preparation, AF, DK, JW, GK, and EL. Writing—review and editing, DJ, LG, DY, and EL. Funding acquisition, EL. All authors have read and agreed to the published version of the manuscript.

## Funding

This research was funded by National Institutes of Health, grant number R15 AI138002.

## Conflict of Interest

The authors declare that the research was conducted in the absence of any commercial or financial relationships that could be construed as a potential conflict of interest.

## Publisher’s Note

All claims expressed in this article are solely those of the authors and do not necessarily represent those of their affiliated organizations, or those of the publisher, the editors and the reviewers. Any product that may be evaluated in this article, or claim that may be made by its manufacturer, is not guaranteed or endorsed by the publisher.

## Supplementary Material

Supplementary File 1 Giemsa-stained smears for *in vitro* maturation of *P. vivax* samples.

Supplementary File 2 Accession numbers for the Ethiopia *P. vivax* transcriptomic data used in gene expression plots.

Supplementary File 3 Nucleotide and haplotype diversity scores of 28 gametocyte genes in the East African, Southeast Asian, and South American *P. vivax* isolates by DNASP analyses.

Supplementary File 4 Phylogenetic trees for two gametocyte genes *GDV1* (PVP01_0734100) and CPW-WPC gene PVP01_1320100 that showed the mix of Ethiopian *P. vivax* together with the Southeast Asian and South American isolates in major clades.

Supplementary File 5 Primer information of two gametocyte genes *Pvs230* (PVP01_0415800) and *PvULG8* (PVP01_1452800).

Supplementary File 6 Pairwise *F*_ST_ matrix and results of AMOVA between all countries of seven targeted gametocyte genes. *P*-values were indicated to show the level of significance.
