# Supplementary material for: Gene Polymorphisms Among Plasmodium vivax Geographical Isolates and the Potential as New Biomarkers for Gametocyte Detection
Source: Front Cell Infect Microbiol. 2022 Jan 13;11:789417. doi: 10.3389/fcimb.2021.789417 (PMC8793628; doi:10.3389/fcimb.2021.789417)
Supplement: Supplementary File 4 — Phylogenetic trees for two gametocyte genes GDV1 (PVP01_0734100) and CPW-WPC gene PVP01_1320100 that showed the mix of Ethiopian P. vivax together with the Southeast Asian and South American isolates in major clades. [file DataSheet_4.docx]

***Pvs230***

Amplicon size = 2552bp

Forward primer_1: GGC TCC TCT TCG GAA GAC GCG TAC TCC GT

Reverse primer_1: TCG GAG GCA CTC GAG CTG ACG GAC AAT ATG GC

Amplicon size = 1084bp

Forward primer_2: GGT GAT GGC GGC GAC TCC TCT ATC CCC ATG G

Reverse primer_2: GAG CCA TGG CGC GCA GGC GGG AGG CAC CTC CGG

***PvULGA***

Amplicon size = 3048bp

Forward primer: GAC CAG TGA GCG GGG CAG GGC TGG GAC CAG T

Reverse primer: CCT AAC AGT GCT GTG CGC GCG CCC C
