# Supplementary material for: Gene Polymorphisms Among Plasmodium vivax Geographical Isolates and the Potential as New Biomarkers for Gametocyte Detection
Source: Front Cell Infect Microbiol. 2022 Jan 13;11:789417. doi: 10.3389/fcimb.2021.789417 (PMC8793628; doi:10.3389/fcimb.2021.789417)

SGH-003
schizonts from thick smear

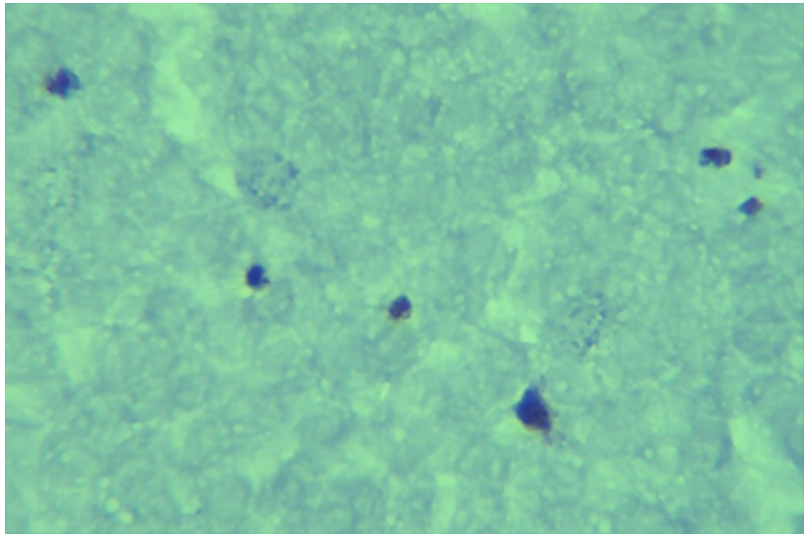

UHC-020
schizont from thick smear

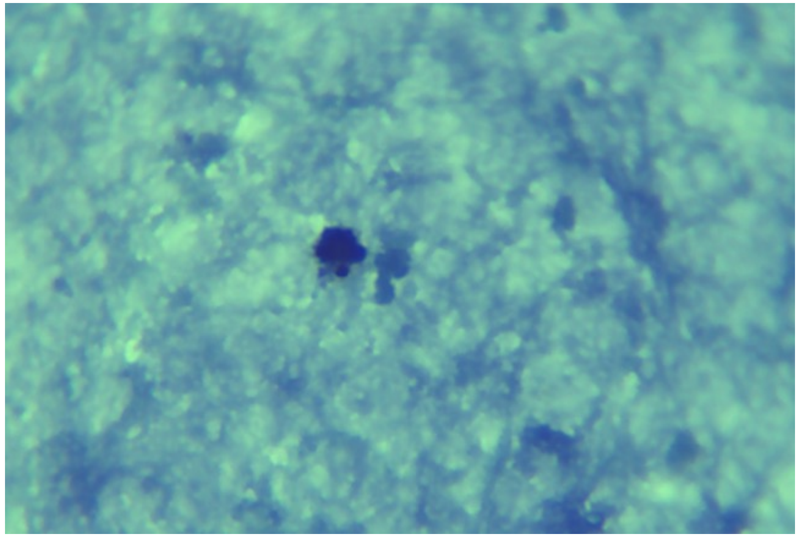

GHC-024
schizonts from thin smear

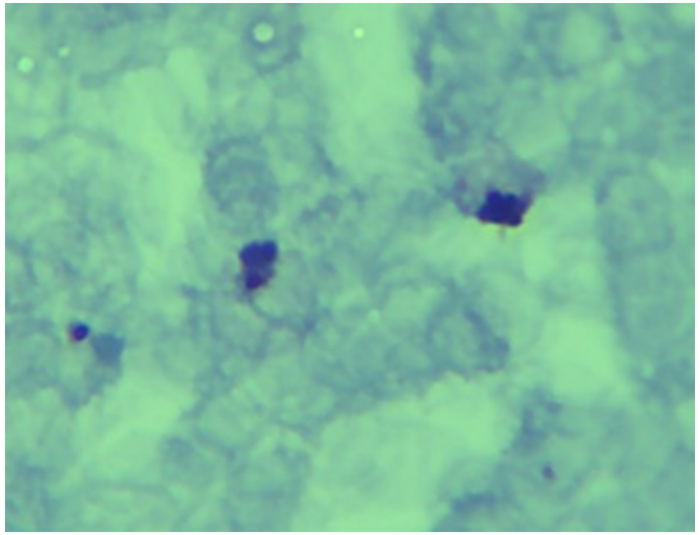

GHC-017
schizont from thin smear

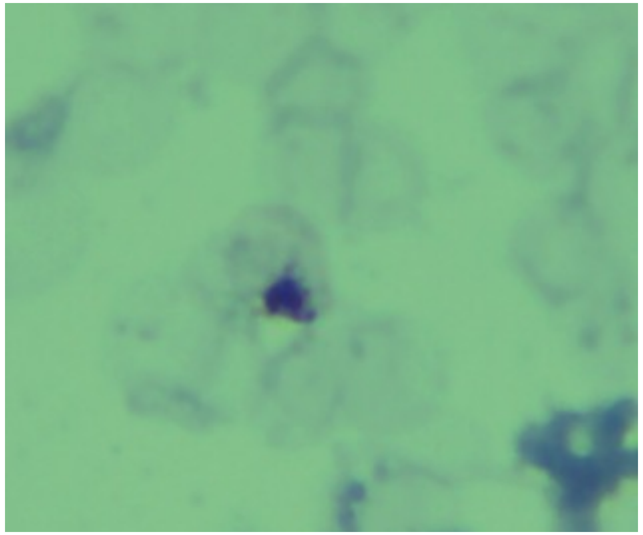

Supplement: Supplementary File 5 — Primer information of two gametocyte genes Pvs230 (PVP01_0415800) and PvULG8 (PVP01_1452800). [file Image_1.pdf]
