# Supplementary figures and images for: Gene Polymorphisms Among Plasmodium vivax Geographical Isolates and the Potential as New Biomarkers for Gametocyte Detection
Source: Front Cell Infect Microbiol. 2022 Jan 13;11:789417. doi: 10.3389/fcimb.2021.789417 (PMC8793628; doi:10.3389/fcimb.2021.789417)

PVP01\_0734100

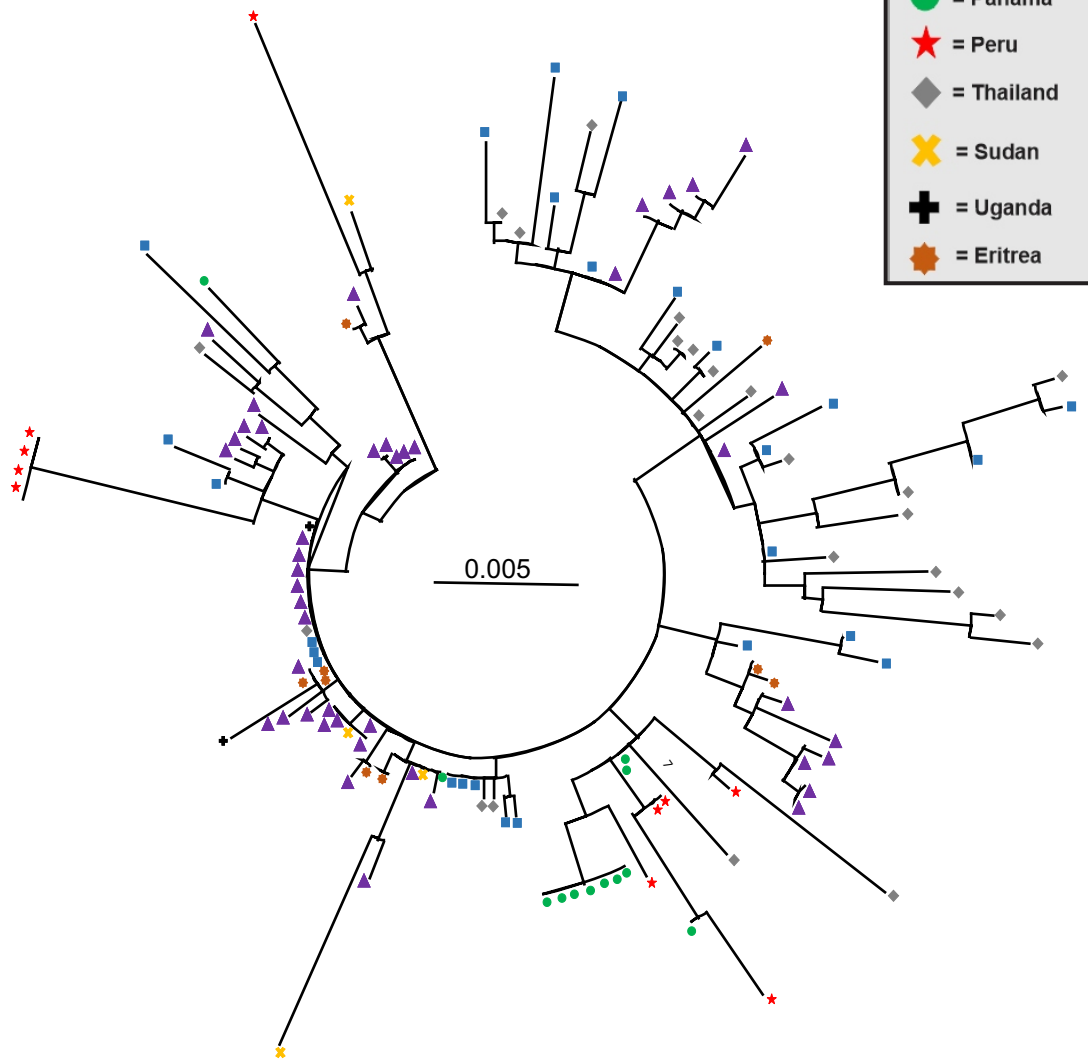

**Legend**

- = Cambodia
- ▲ = Ethiopia
- = Panama
- ★ = Peru
- ◆ = Thailand
- ✕ = Sudan
- ✚ = Uganda
- ⬠ = Eritrea

PVP01\_1320100

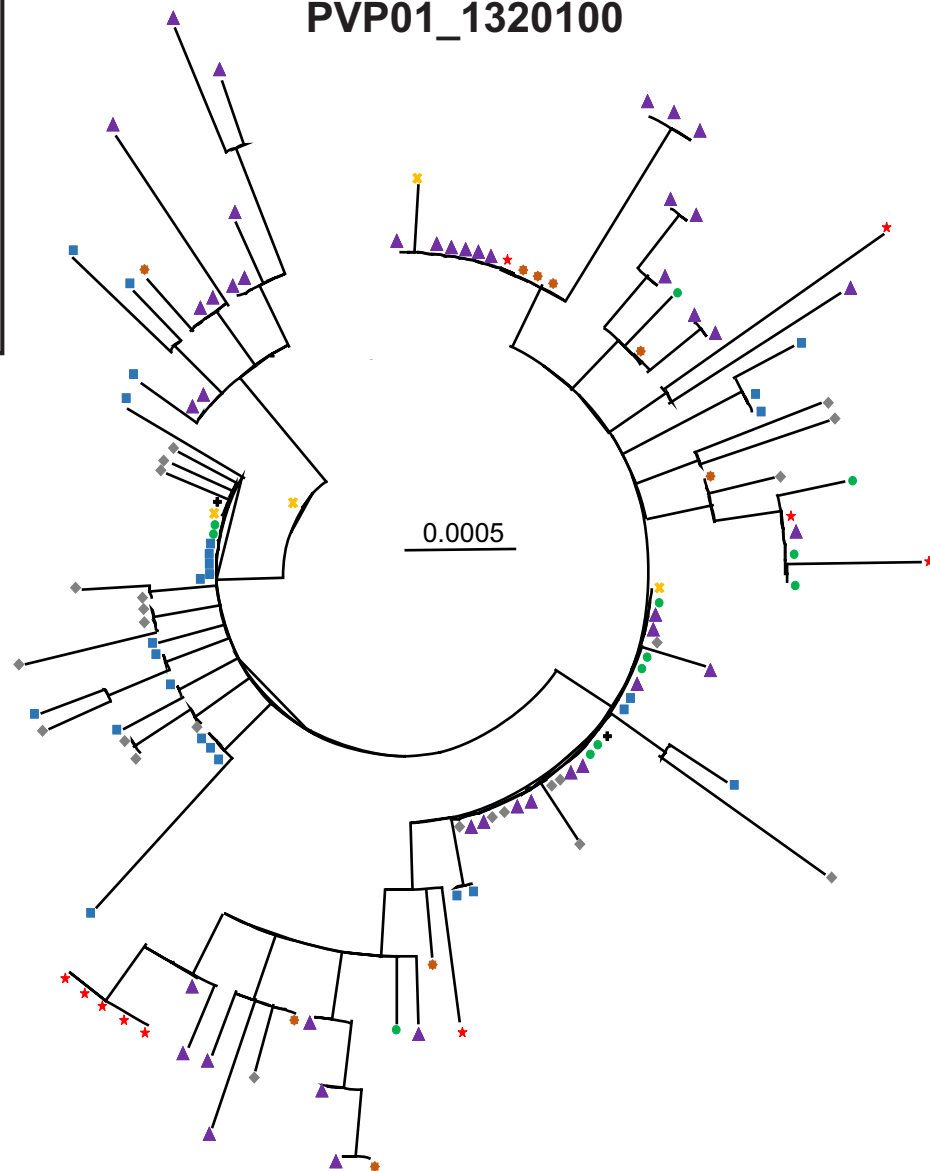

Supplement: Supplementary File 6 — Pairwise F ST matrix and results of AMOVA between all countries of seven targeted gametocyte genes. P-values were indicated to show the level of significance. [file Image_2.pdf]
